# Genome-Wide Analysis of *Serine Carboxypeptidase-like Genes* in Soybean and Their Roles in Stress Resistance

**DOI:** 10.3390/ijms25126712

**Published:** 2024-06-18

**Authors:** Long He, Qiannan Liu, Shaojie Han

**Affiliations:** 1 State Key Laboratory of Rice Biology and Breeding, Key Laboratory of Biology of Crop Pathogens and Insects of Zhejiang Province, Institute of Biotechnology, Zhejiang University, Hangzhou 310058, China; hnndhelong2@163.com (L.H.); lqn053@163.com (Q.L.); 2 Zhejiang Lab, Hangzhou 310058, China

**Keywords:** serine carboxypeptidase-like genes, soybean genome-wide analysis, abiotic and biotic stress, stress resistance

## Abstract

The *serine carboxypeptidase-like* (*SCPL*) gene family plays a crucial role in the regulation of plant growth, development, and stress response through activities such as acyltransferases in plant secondary metabolism pathways. Although *SCPL* genes have been identified in various plant species, their specific functions and characteristics in soybean (*Glycine max*) have not yet been studied. We identified and characterized 73 *SCPL* genes, grouped into three subgroups based on gene structure and phylogenetic relationships. These genes are distributed unevenly across 20 soybean chromosomes and show varied codon usage patterns influenced by both mutation and selection pressures. Gene ontology (GO) enrichment suggests these genes are involved in plant cell wall regulation and stress responses. Expression analysis in various tissues and under stress conditions, including the presence of numerous stress-related cis-acting elements, indicated that these genes have varied expression patterns. This suggests that they play specialized roles such as modulating plant defense mechanisms against nematode infections, enhancing tolerance to drought and high salinity, and responding to cold stress, thereby helping soybean adapt to environmental stresses. Moreover, the expression of specific *GmSCPLs* was significantly affected following exposure to nematode infection, drought, high salt (NaCl), and cold stresses. Our findings underscore the potential of *SCPL* genes in enhancing stress resistance in soybean, providing a valuable resource for future genetic improvement and breeding strategies.

## 1. Introduction

Soybean (*Glycine max*) is one of the most important crops globally, serving as a fundamental component of agriculture by providing essential protein and oil for human and animal consumption [1,2]. Additionally, soybeans are increasingly used in biodiesel production, a renewable energy source that can reduce reliance on fossil fuels [3]. However, various biotic stressors such as pests, diseases, and weeds, along with abiotic stresses including extreme temperatures, drought, and saline conditions, pose significant threats to soybean yield and quality worldwide [4,5,6,7]. In plant biology, serine carboxypeptidases (SCPs) and their closely related serine carboxypeptidase-like proteins (SCPLs) have drawn considerable interest due to their complex roles in plant growth, development, and responses to various stresses [8,9,10,11,12].

Both SCPs and SCPLs are part of the S10 subgroup within the Serine Carboxypeptidase (SCP) family, noted for their highly conserved α/β hydrolase fold. This structural motif includes a central β-sheet surrounded by α-helices on both sides, creating a sturdy globular shape [13,14]. Typically, SCPL proteins contain a conserved triad of amino acids: Serine, aspartate, and histidine (Ser-Asp-His) [14,15]. Most SCPL proteins, characterized by the PF00450 domain in the Pfam database, share structural features that include four evolutionarily conserved domains involved in substrate binding and catalytic activities, a signal peptide for internal transport or secretion, and several N-linked glycosylation sites [16].

SCPs are proteolytic enzymes that facilitate the cleavage of peptide bonds at the carboxyl terminus of proteins and peptides. In contrast, SCPLs, while structurally similar to SCPs, have adapted to engage in a wider array of biochemical processes, notably the modification of secondary metabolites and the regulation of phytohormones. A notable feature of SCPLs is their important role in plant defense mechanisms [17,18]. It was found that the expression of the *OsBISCPL1* gene in rice significantly increased when exposed to defense-related signaling compounds such as salicylic acid (SA) and jasmonic acid (JA) or following bacterial infection [11]. More severe symptoms of Verticillium wilt in cotton (*Gossypium hirsutum*) were displayed when *GhSCPL42* was silenced by virus-induced gene silencing (VIGS) [19]. An increase in proline levels and a reduction in malondialdehyde, leading to enhanced resistance to drought and salt stress, were reported to result from the overexpression of *TaSCPL184-6D* in *Arabidopsis* plants [20].

In this study, we conducted a comprehensive examination of the entire *SCPL* gene family in soybean using the well-established Williams 82 soybean reference genome (https://www.soybase.org/, Wm82.a2.v1), also referred to as Wm82 [21]. We successfully identified and characterized 73 *SCPL* gene family members within the Wm82 soybean genome. This investigation provides new insights into the *SCPL* genes by detailing their gene structures, evolutionary relationships, growth patterns, and expression profiles in soybean. Our results not only facilitate further research on *SCPL* genes in soybean but also contribute to the enhancement of soybean resistance to various stressors through the development of *SCPL* genes.

## 2. Results

### 2.1. Genome-Wide Identification and Analysis of the SCPL Gene Family

*SCPL* genes were examined in various plant species, including *Arabidopsis thaliana*, rice (*Oryza sativa*), and bread wheat (*Triticum aestivum*), as reported by several studies [20,22,23]. However, the specific functions and characteristics of *SCPL* genes in soybean (*Glycine max*) have not been fully explored. The specific locus identifiers and genetic sequences of these genes are comprehensively listed in Supplementary Table S1. A genome-wide search was conducted within the soybean genome using Tbtools to identify all members of the *SCPL* family. Following this, candidates were verified through the protein family database (Pfam) search and NCBI Batch CDD for functional annotations. In soybean, we identified and characterized a total of 73 *serine carboxypeptidase-like* (*SCPL*) genes. These genes were assigned unique identifiers, ranging from *GmSCPL1* to *GmSCPL73*, according to their specific locations on the soybean chromosomes (Table 1). The detailed information about each identified *SCPL* gene in soybean, including open reading frame (ORF) lengths, amino acid lengths, molecular weights (MWs), theoretical isoelectric points (PIs), numbers of introns, splice variants, and subcellular locations, is provided in Table 1. The ORF lengths of the identified *GmSCPLs* ranged from 654 base pairs (*GmSCPL38, Glyma.12G179400.1*) to 1596 base pairs (*GmSCPL40, Glyma.13G028800.1*). The sizes of the proteins varied from 218 to 532 amino acids, with molecular weights ranging from 18.63 kDa to 59.81 kDa, and PI values between 4.81 (GmSCPL63, Glyma.19G128100.1) and 9.17 (GmSCPL23, Glyma.09G249500.1). The count of introns, along with splice variants, varied from 0 to 13 and 1 to 9, respectively. Subcellular localization prediction, using the Plant-mPLoc tool, indicated that most GmSCPLs are located in the vacuole.

### 2.2. Phylogenetic Analysis of the SCPL Family

**Figure 1:**
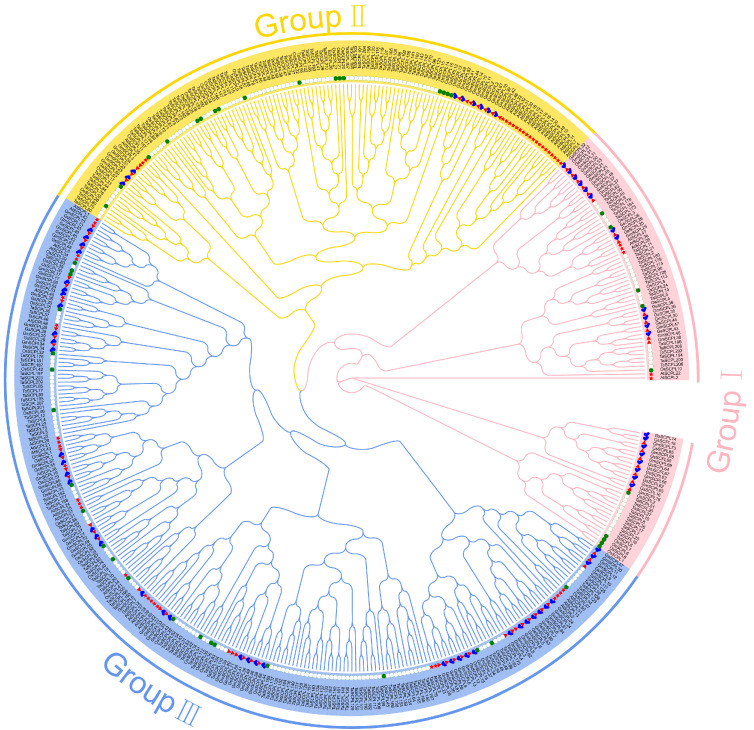
The maximum likelihood (ML) phylogenetic tree was constructed based on the SCPL amino acid sequences in *Arabidopsis thaliana*, *Oryza sativa*, *Triticum aestivum, Glycine max,* and *Glycine soja* using IQ-TREE 2.2.5 software(Q-TREE 2.2.5, Canberra, Australia) with Q. pfam+R6 model. All SCPLs are divided into three phylogenetic groups.

**Figure 2:**
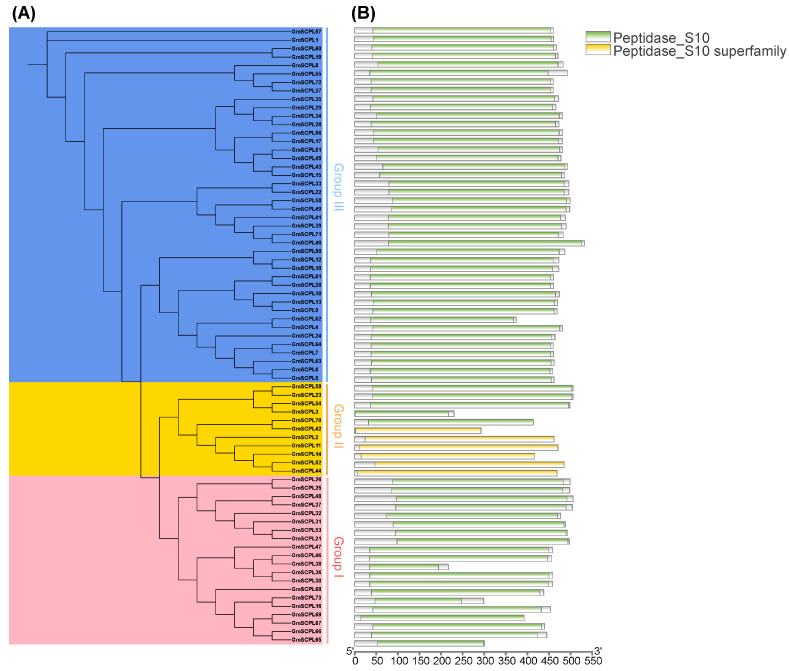
Phylogenetic and conserved domain analysis of the *GmSCPL* family. (**A**) Maximum likelihood (ML) phylogenetic tree of GmSCPLs using IQ-tree with LG+R4 model. All GmSCPLs are divided into three phylogenetic groups. (**B**) Conserved domains of all identified GmSCPLs.

To elucidate the evolutionary relationships among SCPL proteins from GmSCPLs and other well-documented plant SCPLs, we utilized protein sequences from *Arabidopsis thaliana*, rice (*Oryza sativa*), bread wheat (*Triticum aestivum*), soybean (*Glycine max*), and wild soybean (*Glycine soja*). These sequences were analyzed to construct a maximum likelihood phylogenetic tree using the IQ-TREE 2.2.5 software(IQ-TREE 2.2.5, Canberra, Australia), employing the Q.pfam+R6 model (Figure 1). Additionally, a separate phylogenetic tree was generated using only the 73 GmSCPL protein sequences, applying the LG+R4 model to further refine our understanding (Figure 2A). NCBI Batch CDD analysis confirmed that each GmSCPL sequence contained one or two conserved S10 regions (Figure 2B). The identified GmSCPLs were classified into three distinct subgroups:Ⅰ, Ⅱ, and Ⅲ, consistent with several previous studies [19,20,23].

### 2.3. Chromosomal Distribution and Gene Duplication of the GmSCPL Family

**Figure 3:**
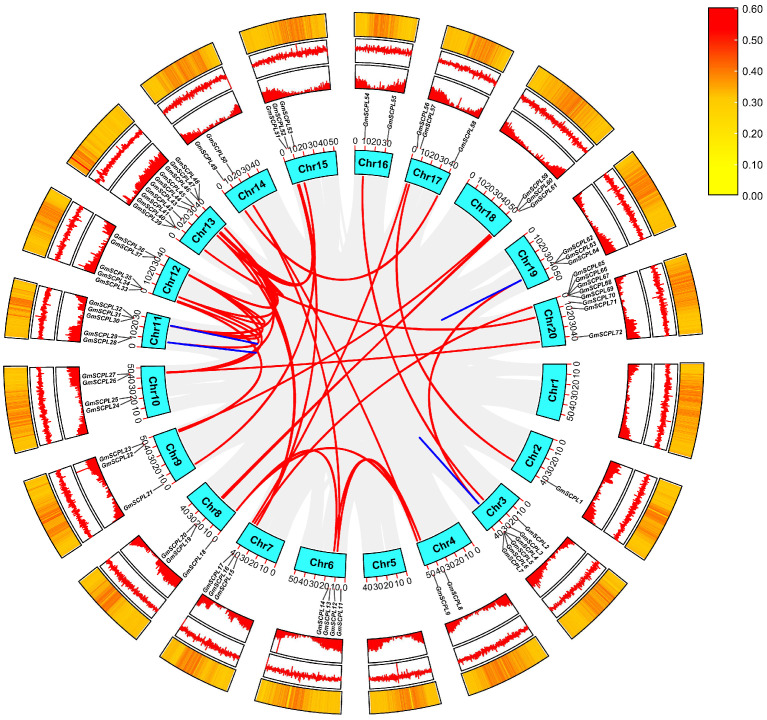
Chromosome location and gene duplication analysis of *GmSCPL* genes. Slight gray lines indicate all synteny blocks within soybean genome. The duplicated gene pairs are highlighted with colored lines. Heat map in the inner circle indicates chromosomal gene density; Heat map in the middle circle indicates chromosomal GC skew; Heat map in the outer circle indicates chromosomal GC contents.

To investigate the chromosomal distribution characteristics of the *GmSCPL* family, we analyzed their locations across soybean chromosomes. Figure 3 and Figure S1 show that chromosome 13 harbors the highest number of *GmSCPL* genes. In contrast, chromosome 2 contains only one *GmSCPL* gene, highlighting an uneven distribution of the 73 *GmSCPLs* across the 20 chromosomes of soybean. The distribution pattern of *SCPL* genes across the chromosomes does not exhibit a strong correlation with gene density, GC content, or GC skew (Figure 3).

The soybean cyst nematode (SCN) is recognized as a significant global threat to soybean production [4,24]. Notably, our analysis revealed that 38.35% (28 out of 73) of the identified *GmSCPL* genes are located within regions corresponding to known SCN-related quantitative trait loci (QTLs), as shown in Figure S1 and Table S2. Additionally, RNA-seq analysis indicated that the expression of these *SCPL* genes was significantly induced 5 days post-inoculation with SCN (Figure S2). While this study provides a comprehensive identification and characterization of *SCPL* genes, future research will focus on real-world verification and functional studies to further elucidate the roles of these genes in soybean’s defense mechanisms against SCN.

### 2.4. Analysis of SCPL Paralogs and Orthologs

**Figure 4:**
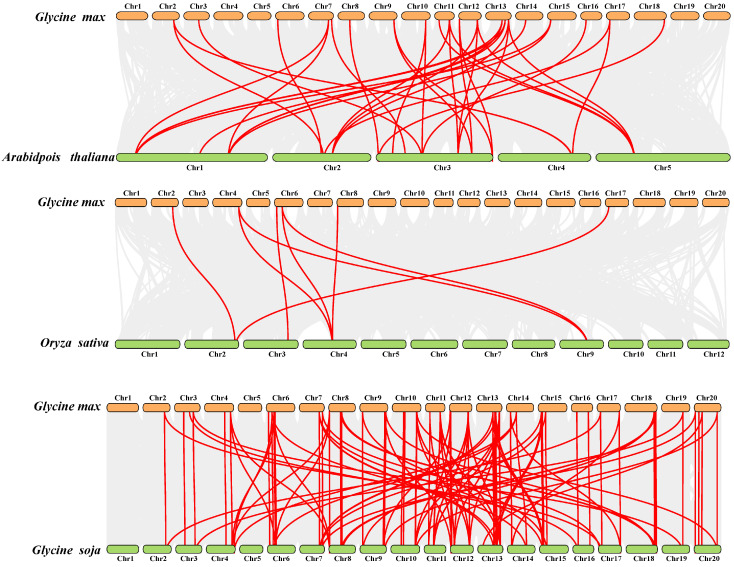
Syntenic relationships of the *SCPL* genes in *Glycine max* and three other species, including *Arabidopsis thaliana*, *Oryza sativa,* and *Glycine soja*.

To delve deeper into uncovering the evolutionary links within the *SCPL* gene family, we conducted a syntenic analysis aimed at identifying *SCPL* paralogous gene pairs within soybean (*G. max*) and *SCPL* orthologous gene pairs between soybean and monocotyledonous plants (*O. sativa*) as well as dicotyledonous plants (*Arabidopsis* and *G. soja*) using Multiple Collinearity Scan Toolkit (MCScanX, Athens, Greece). We identified 40 paralogs in soybean (*Gm*-*Gm*) (Table S3). Among these, 33 orthologous gene pairs were detected between *Arabidopsis* and soybean (*Gm*-*At*), 117 orthologous gene pairs between *G. soja* and soybean (*Gm*-*G. soja*), and only eight SCPL orthologous gene pairs between rice and soybean (*Gm*-*Os*) (Figure 4). These findings suggest a close linkage between *SCPL* genes in soybean and those in *G. soja*, possibly due to their close genetic relationship [25,26,27].

The Ka/Ks ratio, a crucial parameter reflecting selective pressure on plant genes, was utilized to assess evolutionary patterns [28]. The Ka (non-synonymous substitution rate) measures the rate of amino acid-changing mutations, while the Ks (synonymous substitution rate) measures the rate of mutations that do not change the amino acid sequence. The Ka/Ks ratio indicates the type of selective pressure acting on a gene: A ratio greater than 1 suggests positive selection (adaptive evolution), a ratio equal to 1 suggests neutral evolution, and a ratio less than 1 suggests purifying selection (removal of deleterious mutations) [29]. Divergence time (T) was calculated based on Ks values to estimate the evolutionary time scale of gene duplications. For each *SCPL* gene pair, we calculated the Ka, Ks, Ka/Ks, and T values. Our analysis revealed that the Ka/Ks ratios of all paralogous genes (*Gm-Gm*) and most orthologous genes *(Gm-G. soja*, *Gm-Os*, and *Gm-At*) were below 1 (see Tables S3 and S4), indicating that purifying selection plays a dominant role in the evolution of the *SCPL* family. Additionally, the divergence time (T) of the 40 paralogs (*Gm-Gm*) ranged from 2.03 to 58.61 million years ago (Mya) (Table S3). The divergence time of orthologs (*Gm-G. soja*) ranged from 0.09 to 58.6 Mya, while it ranged from 51.41 to 152.16 Mya in *Gm-At* and from 69.51 to 74.02 Mya in *Gm-Os*. These results further underscore the closer genetic relationship between soybeans and wild soybeans (*G. soja*) on an evolutionary scale [30]. It is important to note that these divergence times are specific to the evolutionary history of the *SCPL* gene family within these plant lineages.

### 2.5. Analysis of Codon Usage Patterns in SCPL Genes across Plant Species

Each codon uniquely specifies an amino acid, and multiple codons can encode the same amino acid, termed synonymous codons [31,32]. The frequency of usage for these synonymous codons varies significantly among different species and even within different genes, underscoring the importance of codon preference in evolutionary dynamics [33]. We examined codon usage bias (CUB) across five species, including *Clycine max*, *Clycine soja*, *Oryza sativa*, *Arabidopsis thaliana,* and *Triticum aestivum*, utilizing identified *SCPL* CDS sequences, with detailed information provided in Table S5. Our findings revealed that the average GC content and GC3s of *SCPL* genes ranged from 0.422164384 to 0.575114286 and 0.3610685 to 0.7024667, respectively, across the five species. Notably, the average effective number of codons (ENC) of *SCPL* genes in monocotyledonous species (*O. sativa* and *T. aestivum*) was lower compared to that in dicotyledonous ones (*G. max*, *G. soja*, and *Arabidopsis*) (Table 2), indicating stronger codon usage bias in monocotyledonous plants.

**Figure 5:**
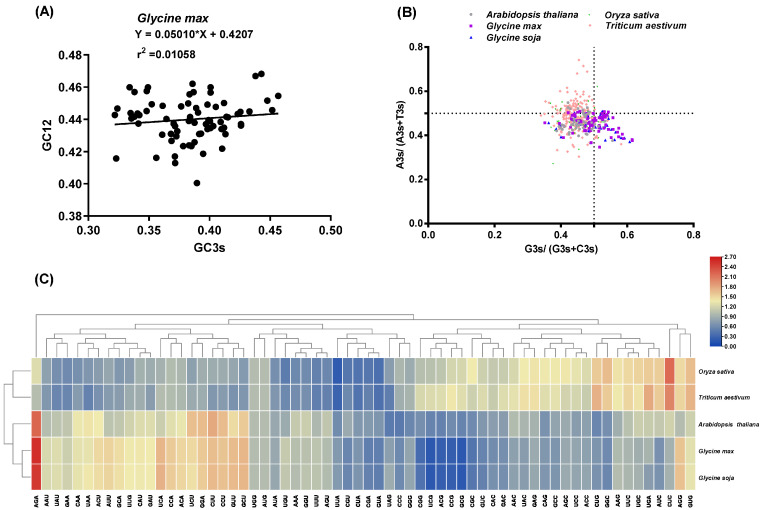
Condon usage bias analysis. (**A**) Neutrality plot analysis of all identified *GmSCPLs* CDS sequences. (**B**) Parity rule 2 (PR2) analysis of *GmSCPLs* CDS sequences in five species including *Arabidopsis thaliana*, *Oryza sativa*, *Triticum aestivum*, *Glycine max*, and *Glycine soja*. (**C**) Heatmap showing relative synonymous codon usage (RSCU) values of five species including *Arabidopsis thaliana*, *Oryza sativa*, *Triticum aestivum* and *Glycine max* and *Glycine soja*. Blue-to-red color indicates low-to-high RSCU values of codons.

Analysis of relative synonymous codon usage (RSCU) provided insights into CUB [34]. We observed that the RSCU patterns of *SCPL* genes in *G. max* and *G. soja* were similar, whereas those in *O. sativa* and *T. aestivum* were clustered (Figure 5C), potentially reflecting their natural evolutionary relationships. Parity rule 2 (PR2) analysis explored GC and AT composition bias at the third codon position [35]. Investigation of PR2 revealed varying ratios of G3s/(G3s+C3s) and A3s/(A3s+T3s) among different species, with most identified SCPL members in the five species preferring C and T over G and A (Figure 5B). Neutral plot analysis confirmed additional factors influencing codon preference beyond base composition. Relatively slight positive correlations between GC3s and GC12 of *SCPL* gene CDS sequences were observed in *G. max* (r^2^ = 0.01058, *p* < 0.01) (Figure 5A), *G. soja* (r^2^ = 0.01346, *p* < 0.01) (Figure S3A), and *Arabidopsis* (r^2^ = 0.4409, *p* < 0.01) (Figure S3B), indicating that both mutation pressure and natural selection influenced the codon preference of *SCPLs* in dicots (*G. max*, *G. soja*, and *Arabidopsis*). Interestingly, notably positive correlations between GC3s and GC12 of *SCPL* gene CDS sequences were evident in rice (r^2^ = 0.7020, *p* < 0.01) (Figure S3C) and bread wheat (r^2^ = 0.6118, *p* < 0.01) (Figure S3D), likely attributable to their monocot classification.

### 2.6. Analysis of Conserved Motifs and Gene Structure in GmSCPLs

**Figure 6:**
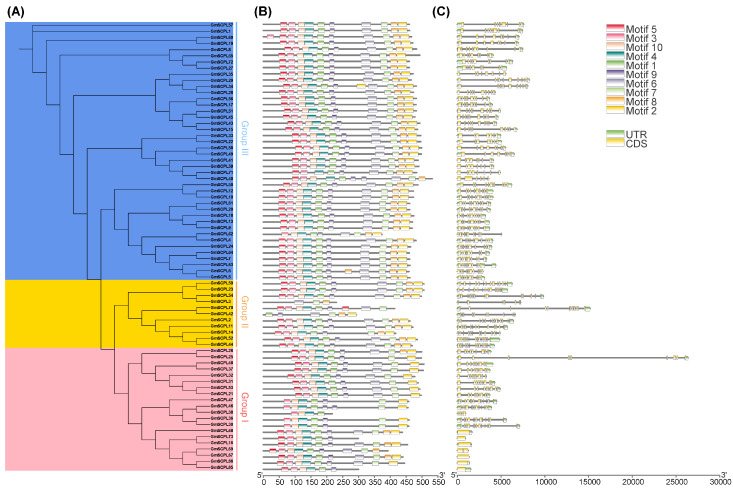
Analysis of the phylogenetic relationship, gene struchure and conserved motfs of GmSCPLs. (**A**) Phylogenetic tree of GmSCPLs. (**B**) Conserved motifs of GmSCPLs. Different colors indicate different motifs numbered 1 to 10. (**C**) Gene structure of all identified *GmSCPLs*. Green boxes indicate untranslated regions (UTRs), and yellow boxes indicate coding sequence (CDS) regions.

Examining the exon–intron architecture of genes provides valuable insights into their evolutionary paths and functional diversification. We conducted an analysis of the gene structure and conserved motifs of *GmSCPLs*, revealing a range of exon numbers from 1 to 14 (Table 1). Notably, this analysis highlights a degree of conservation in exon–intron structures within each branch of the evolutionary tree (Figure 6C).

To identify potential motifs within the soybean *SCPL* gene family, we subjected the deduced amino acid sequences of 73 GmSCPL proteins to motif prediction analysis using the MEME suite. As illustrated in Figure 6B, ten conserved motifs (designated as Motif1-10) were predicted and visualized across all identified GmSCPLs. The distribution of motifs among the GmSCPLs varied, with counts ranging from three to ten. Remarkably, certain motifs were prevalent among most GmSCPLs, while others were specific to particular clusters. For example, Motifs 1, 3, 4, 5, 9, and 10 were consistently found at the 5′ terminal of 98% of identified GmSCPL members, whereas motif 2 or 8 existed at the 3′ terminal of 95.8% of identified GmSCPL members (Figure 6B). Additionally, we observed that GmSCPLs within the same subgroup exhibited a similar order of motif distribution, suggesting potential functional homogeneity.

### 2.7. Prediction of cis-Acting Regulatory Elements in Promoter Regions of GmSCPLs

**Figure 7:**
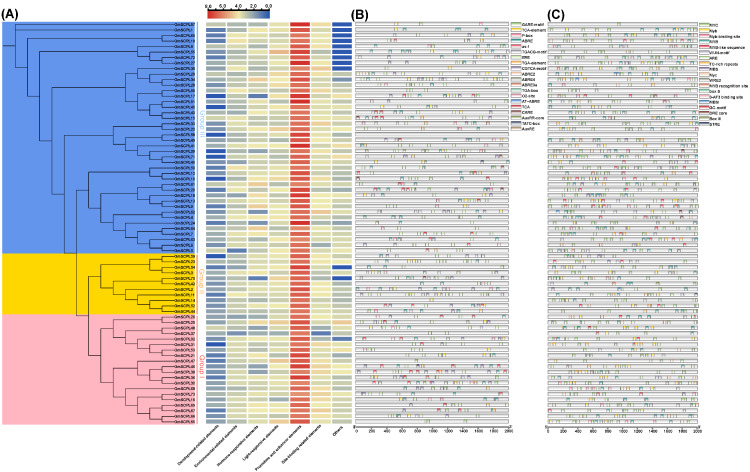
Analysis of cis-acting regulatory elements in *GmSCPLs*. (**A**) The number of seven distinct types of cis-acting elements within the promoter regions of each *GmSCPL.*; (**B**) overview of environmental-related cis-acting regulatory elements in each *GmSCPL.;* (**C**) overview of hormone-relate cis-acting regulatory elements in each *GmSCPL*.

In the realm of plants, a subset of cis-acting regulatory elements holds considerable importance in orchestrating responses to diverse stressors, such as hormonal fluctuations, dehydration, cold, and light exposure, through the binding of specific transcription factors [36,37,38,39,40,41]. Furthermore, certain cis-regulatory elements have been identified as pivotal mediators of plant immune mechanisms [42,43,44,45]. To explore the expression patterns of *GmSCPL* genes, we screened their 2.0 kb promoter region sequences using the PlantCARE database [46]. A total of 12,469 cis-acting regulatory elements were predicted and identified, categorized into groups related to development, environmental stress response, hormone signaling, light responsiveness, and promoter and site-binding elements (Figure 7A). This underscores the significant role of cis-acting elements in governing *SCPL* gene expression during soybean growth and development. As depicted in Figure 7B,C, a total of 590 environmental-related elements and 722 hormone-related elements were characterized, suggesting that *GmSCPL* genes may not only impact soybean growth and development but also play a profound role in regulating and responding to various biotic and abiotic stresses.

### 2.8. Gene Ontology (GO) Enrichment Analysis of GmSCPLs

**Figure 8:**
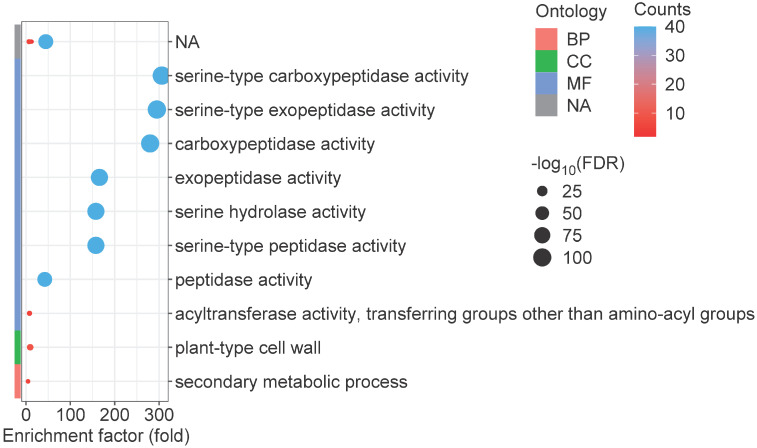
Gene Ontology (GO) enrichment analysis of *GmSCPL* genes, organized into three categories: biological process (BP), cellular component (CC), and molecular function (MF). GO terms with a *p*-value below 0.05 were deemed statistically significant, indicating notable enrichment.

Gene Ontology (GO) enrichment analysis is commonly employed as a fundamental approach for elucidating gene functionality [47]. Consequently, we conducted GO enrichment analysis of all identified *GmSCPL* members. *GmSCPLs* within the biological processes (BP) category were enriched in secondary metabolic process; those within the cellular components (CC) category were associated with plant-type cell wall; while those within the molecular function (MF) category were primarily enriched in serine-type peptidase activity, serine-type exopeptidase activity, serine-type carboxypeptidase activity, exopeptidase activity, serine hydrolase activity, and peptidase activity (Figure 8). These findings suggest that the *GmSCPL* family may be involved in the stress response and regulation of plant cell wall dynamics.

### 2.9. Analysis of Tissue-Specific Expression Patterns of Selected GmSCPLs

**Figure 9:**
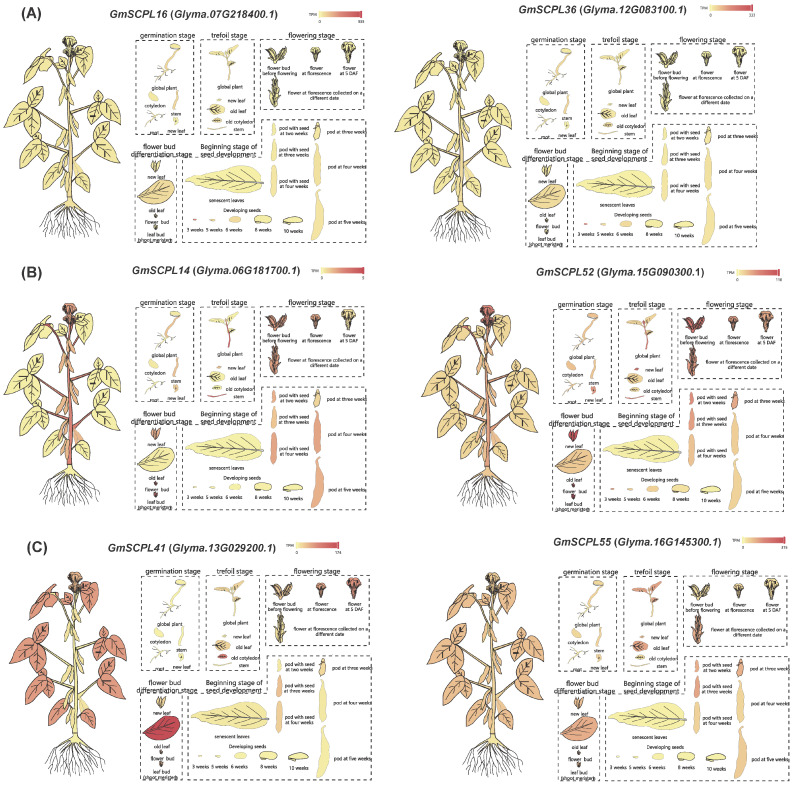
Differential expression of selected *GmSCPLs* in different tissues by RNA-seq. (**A**) Expression profiles of *GmSCPL16* (*Glyma.07G218400.1*) and *GmSCPL36* (*Glyma.12G083100.1*) across various soybean tissues at different developmental stages. (**B**) Expression profiles of *GmSCPL14* (*Glyma.06G181700.1*) and *GmSCPL52* (*Glyma.15G090300.1*) across various soybean tissues at different developmental stages. (**C**) Expression profiles of *GmSCPL41* (*Glyma.13G029200.1*) and *GmSCPL55* (*Glyma.16G145300.1*) across various soybean tissues at different developmental stages. The heatmaps indicate the expression levels, with red representing high expression and yellow representing low expression. Each diagram illustrates the expression patterns in tissues such as roots, stems, leaves, flowers, and pods, providing a comprehensive overview of the spatial and temporal expression of these genes.

To explore the functions of *GmSCPLs*, we analyzed the transcriptional levels of six specific *GmSCPL* genes in various tissues using RNA-seq Tools of SoyMD [48]. These genes included two from each subgroup (Ⅰ, Ⅱ, and Ⅲ) and were all located within SCN-related QTLs (soybean cyst nematode-related quantitative trait loci) (Figure S1 and Table S2). We measured expression levels in terms of transcripts per kilobase million (TPM). Figure 9 shows that each *GmSCPL* was transcriptionally active in at least one specific organ. Notably, while the expression patterns varied among different phylogenetic subgroups, they were quite consistent within each subgroup. This indicates that *GmSCPLs* may have important roles in tissue-specific developmental processes at various stages of growth.

### 2.10. Expression Analysis of GmSCPLs under Abiotic and Biotic Stress

**Figure 10:**
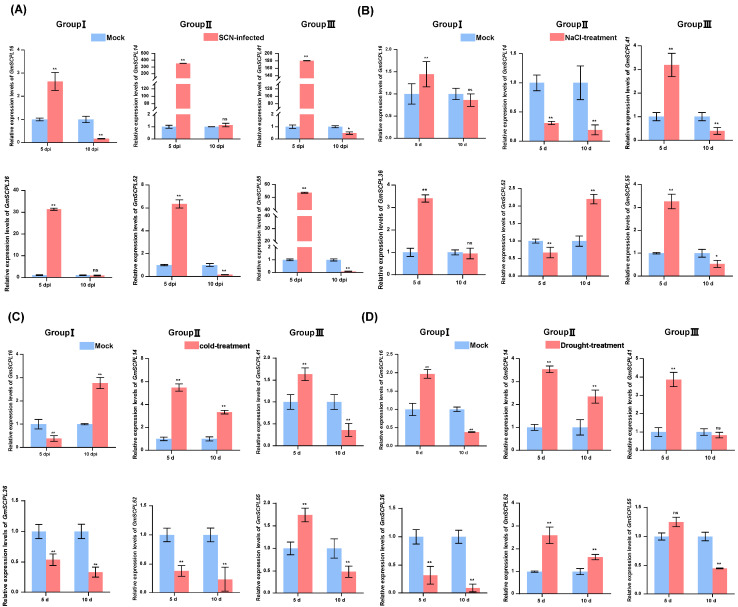
Analysis of the expression of certain *GmSCPLs* in response to biotic and abiotic stresses. (**A**) Expression profiles of certain *GmSCPLs* in response to soybean cyst nematode (SCN, *Heterodera glycines*) infection were analyzed by RT-qPCR. The mean values and standard errors (SE) were calculated from three biological replicates, each consisting of three technical replicates: ns indicates non-significance; ** *p* < 0.01; * *p* < 0.05 (Student’s *t*-test). (**B**) Expression profiles of certain *GmSCPLs* in response to 0.9% NaCl treatment were analyzed by RT-qPCR. Mean values and standard errors (SE) were calculated from three biological replicates, each consisting of three technical replicates. Note: “ns” indicates non-significance; “**” *p* < 0.01; “*” *p* < 0.05 (Student’s *t*-test). (**C**) Expression profiles of certain *GmSCPLs* in response to 4 ℃ treatment were analyzed by RT-qPCR. The mean values and standard errors (SE) were calculated from three biological replicates, each consisting of three technical replicates: “**” *p* < 0.01 (Student’s *t*-test). (**D**) Expression profiles of certain *GmSCPLs* in response to drought treatment were analyzed by RT-qPCR. The mean values and standard errors (SE) were calculated from three biological replicates, each consisting of three technical replicates: “ns” indicates non-significance; “**” *p* < 0.01 (Student’s *t*-test).

Previous studies have established that *SCPLs* in plants are crucial for various physiological and biochemical functions, and their transcription is influenced by a range of stimuli, including pathogenic attacks and environmental stresses such as drought, salt, and cold [19,20,49]. To investigate the roles of *GmSCPLs* under biotic and abiotic stress conditions, we analyzed how soybean cyst nematode (SCN, *Heterodera glycines*), cold, drought, and saline-alkali stresses affect the transcription levels of selected *GmSCPL* genes. Our data show significant up-regulation of all selected genes five days after inoculation with SCN (5 dpi) (Figure 10A). Ten days post-SCN inoculation, *GmSCPL16*, *GmSCPL52*, *GmSCPL41,* and *GmSCPL55* showed significant expulsion, whereas *GmSCPL14* and *GmSCPL36* exhibited no significant changes (Figure 10A). Under saline-alkali or cold stress, *GmSCPL41* and *GmSCPL55* (Group Ⅲ) displayed a strong induction on the fifth day and suppression on the tenth day (Figure 10B,C). Regarding drought stress, the expression of *GmSCPL41* was significantly induced, while *GmSCPL55* displayed no significant change (Figure 10D). The response of Group Ⅰ and Ⅱ members varied significantly under different abiotic stresses. For example, *GmSCPL16* and *GmSCPL36* (Group Ⅰ) showed strong induction under saline-alkali stress on the fifth day, but their expression was not significantly changed under the same condition on the tenth day (Figure 10B). As for *GmSCPL14* and *GmSCPL52* (Group Ⅱ), their expression was vitally suppressed under saline-alkali stress while induced under drought stress on the fifth day (Figure 10B,C). These findings suggest that *GmSCPLs* play significant roles in response to nematode infection and various environmental stresses.

## 3. Discussion

The *serine carboxypeptidase-like genes* (*SCPLs*) hold a significant position in plant growth, development, and responses to various stress [11,50,51,52]. Genome-wide investigation of *SCPLs* in multiple plants, including *Arabidopsis thaliana*, rice (*Oryza sativa*), bread wheat (*Triticum aestivum*), cotton (*Gossypium hirsutum*), rapeseed (*Brassica napus*), and the tea plant (*Camellia sinensis*), had been performed previously [19,20,22,53,54]. As one of the most important protein feed and vegetable oil crops for human consumption, cultivated soybean (*Glycine max*) originated from wild soybean (*Glycine soja*) in China about 5000 years ago [26,27,55]. Williams 82 (Wm82) was the first-released reference genome among plenty of cultivated soybeans [56,57]. Nevertheless, a systemic analysis of the *SCPL* gene family in soybean has been limited until now. Here, 73 *SCPL* members were identified and characterized based on the reference genome of Wm82. All identified GmSCPLs could be stratified into three distinct clusters (termed Group Ⅰ, Ⅱ, and Ⅲ) on the bias of their gene structure and phylogenetic analysis (Figure 2A and Figure 3), in accordance with several earlier observations [19,20,23]. Our analysis revealed that the conserved domain, motif, and gene structure of most identified GmSCPLs were relatively conserved within the same cluster (Figure 2 and Figure 6). Additionally, most members of the identified GmSCPLs were predicted to be localized in the vacuole or peroxisome (Table 1), which might be due to the indispensable role SCPLs played in the presence of plant secondary metabolites [58,59].

The number of *SCPLs* varies a lot across different species (Table 1 and Table S1). For instance, bread wheat (*T. aestivum*) owns the largest number (210) of *SCPL* gene family members, which can be attributed to the fact that wheat is a complex allohexaploid and has experienced two whole genome duplications. The number of *SCPL* members in soybean (*G. max*) and wild soybean (*G. soja*) is quite comparable (Table 1 and Table S1). Furthermore, 117 orthologous gene pairs were identified between soybean and wild soybean (*G. max*-*G. soja*), much more abundant than those in other species (Figure 4 and Table S4). These observations may be due to the fact that *G. max* and *G. soja* are closely related wild relatives [26,27,60]. Gene duplication events are fundamental for the gene family, and they can facilitate plants in nature becoming more and more adapted to diverse environmental conditions [61,62,63]. Our exploration revealed that six tandem duplication events and 34 fragment duplication events contributed to the *SCPL* gene family in soybean (Figure 3), suggesting both fragment and tandem duplication events were vital for *GmSCPLs*’s expansion.

It is highly frequent for codon usage bias (CUB) to occur within the plant genome [64,65]. CUB occupies a significant position in the regulation of gene expression and molecular evolution [66,67]. CUB, including codon bias index (CBI), frequency of optimal codons (Fop), effective number of codons (ENC), GC content at the third position (GC3s), and overall GC content of *SCPL* gene family analysis, was performed in soybean. Taking these CUB indicators into account, we found that the CUB of *SCPL* genes in dicotyledonous plants (*G. max*, *G. soja,* and *Arabidopsis*) was more intense than that in monocotyledonous ones (*O.sativa* and *T. aestivum*) (Table 2). We also examined the relative synonymous codon usage (RSCU) values of the *SCPL* gene family across five species. A relatively conserved RSCU pattern within monocotyledons or dicotyledons was also observed (Figure 5C). Furthermore, neutral plot and parity rule 2 (PR2) analysis showed that CUB of the *SCPL* gene family in monocotyledonous plants is mainly influenced by natural selection, while both mutation and natural pressure function in dicotyledonous ones (Figure 5A,B).

The *SCPL* gene family exhibits a broad spectrum of expression across different tissues [19]. RNA-seq analysis revealed that all selected *GmSCPLs* exhibited higher expression levels in the upper flower, indicating their potential involvement in plant growth and development (Figure 9). The *GmSCPLs* promoter analysis identified a series of cis-acting elements, encompassing those elements related to development, environmental stress, and hormone responsiveness (Figure 7), which suggests *GmSCPLs* may play an important role in soybean response to abiotic or biotic stresses. Then, we explored the expression of *GmSCPLs* under biotic stress (nematode infection) and abiotic stress (NaCl, cold, and drought treatments). As shown in Figure 10, the outcomes revealed that the expression levels of all selected *GmSCPLs* were significantly influenced by abiotic or biotic stress. These results suggest that GmSCPLs may have distinct functions in combating various natural stresses.

Through a blend of genomic, bioinformatic, and experimental approaches, this study lays a solid foundation for future functional studies and genetic engineering efforts aimed at exploiting the potential of *SCPL* genes to improve stress tolerance in soybean. Further research into the specific roles of individual *SCPL* genes will elucidate their contributions to soybeans’ adaptive responses and facilitate the development of targeted breeding strategies for stress resistance.

## 4. Materials and Methods

### 4.1. Identification and Characterization of the SCPL Family in Soybean

For this study, Williams 82 soybean seeds were obtained from the Chinese Academy of Agricultural Sciences in Beijing, China. Reference genomic sequences for *Oryza sativa* (*Oryza sativa v7.0*), *Glycine max* (*Wm82*.*a2*.*v1*), *Arabidopsis thaliana* (*Arabidopsis thaliana TAIR10*), and *Glycine soja* (*G. soja v1.1*) were obtained from the Phytozome Plants database (https://phytozome-next.jgi.doe.gov/, accessed on 6 January 2024). Reference genomic sequences for *Triticum aestivum* (bread wheat) were downloaded from the Ensemble Plants database (http://plants.ensembl.org/Triticum_aestivum/Info/Index; accessed on 6 January 2024). *SCPL* sequences from *A. thaliana* and *O. sativa* were used to perform a local BlastP analysis against the *G. max* (Williams 82) genome with a significance threshold E-value of less than 1 × 10^−10^. The SCPL family HMM profile (PF00450) was downloaded from the PFAM database (http://pfam.xfam.org/; accessed on 6 January 2024) and used for functional annotation with the HMMER software (version 3.0)( HMMER3, Cambridge, UK) [68]. The structural integrity of the SCPL domain in each candidate protein sequence was verified using the NCBI Batch Web CD-Search Tool (https://www.ncbi.nlm.nih.gov/Structure/bwrpsb/Bwrpsb.cgi; accessed on 16 January 2024). In total, 73 *GmSCPL* members were identified. Detailed information on the *GmSCPL* family, including gene locations, open reading frame (ORF) lengths, intron numbers, splice variants, and molecular sizes, was gathered from the Phytozome Plants database. Theoretical isoelectric points (PI) and molecular weights (MW) for the GmSCPLs were calculated using the ExPAsy tool (https://web.expasy.org/compute; accessed on 6 January 2024) [69]. Subcellular localization predictions for the 73 GmSCPLs were made using the Plant-mPLoc tool [70].

### 4.2. Phylogenetic Analysis

Multiple sequence alignments of GmSCPL proteins from *G. max*, *G. soja*, *Arabidopsis*, *O. sativa*, and *T. aestivum* were carried out using ClustalW. Following this, a maximum likelihood (ML) phylogenetic tree was constructed with IQ-Tree 2.2.5 software (IQ-TREE2.2.5, Canberra, Australia) [71] and refined using the iTOL online tool (https://itol.embl.de; accessed on 19 January 2024).

### 4.3. Chromosome Location and Collinearity Analysis

Synteny relationships within the *SCPL* family across *G. max*, *G. soja*, *Arabidopsis*, and *O. sativa* were explored using McscanX [72] and visualized with TBtools [73]. The ratio of nonsynonymous to synonymous substitutions (Ka/Ks) for tandem duplications was determined using the Ka/Ks Calculator in TBtools, and divergence times (T) were calculated with the formula T = Ks/(2 × 9.1 × 10^−9^) million years ago (Mya) [29,74].

### 4.4. Codon Usage Bias Analysis

Codon usage bias for *SCPL* coding sequences (CDS) from *G. max*, *G. soja*, *Arabidopsis*, *O. sativa*, and *T. aestivum* was assessed using CodonW 1.4.2 software (CodonW 1.4.2, Houston, USA). The frequency of optimal codons, effective number of codons (ENC), GC content, and GC3s content, along with relative synonymous codon usage (RSCU), were estimated using the EMBOSS online tool.

### 4.5. Motif and Gene Structure Analysis

Conserved motifs within the GmSCPLs were predicted using the MEME sequence analysis tool (https://meme-suite.org/meme/) with a maximum of ten motifs per sequence [75]. The results were visualized using TBtools software (TBtools, Guangzhou, China) [74]. Additionally, the advanced gene structure view function of TBtools was utilized to analyze and display the gene structures of *GmSCPL* genes [74].

### 4.6. Promoter Analysis

Promoter regions, extending 2000 base pairs upstream from the transcription start sites of 73 *GmSCPL* genes, were extracted from the soybean genome GTF file. These sequences were analyzed using the PlantCARE website (http://bioinformatics.psb.ugent.be/webtools/plantcare/html/; accessed on 6 January 2024) to predict potential cis-acting regulatory elements.

### 4.7. Gene Ontology (GO) Enrichment Analysis

GO annotation enrichment analysis for *GmSCPLs* was performed using the GO Enrichment function of TBtools software (TBtools, Guangzhou, China), which involved transforming the *p*-values using the -log10 conversion [74]. The results were subsequently visualized using the clusterProfiler tool [76].

### 4.8. RNA Isolation and Real-Time Quantitative Polymerase Chain Reaction (RT-qPCR)

Total RNA was isolated following the manufacturer’s instructions using TRIzol Reagent (Vazyme, Nanjing, China). Single-stranded cDNA was synthesized using a First Strand cDNA Synthesis Kit (Vazyme, Nanjing, China). Real-time quantitative PCR (RT-qPCR) was performed using an ABI QuantStudio5 Detection System (Applied Biosystems, Foster City, CA, USA) with Hieff qPCR SYBR Green Master Mix (YEASEN, Shanghai, China). Each experimental condition included at least three biological replicates and three technical replicates. The relative expression levels of target genes were quantified using the 2^−ΔΔC(t)^ method as described by [77]. The *actin* gene served as the internal control for each assay. Primers involved in RT-qPCR are shown in Table S6.

### 4.9. Tissue Expression by RNA-seq

Tissue expression analysis was performed using RNA-seq data obtained from the Soybean multi-omics database (SoyMD) (Soybean: Home (hzau.edu.cn); accessed on 6 January 2024) [48]. The RNA-seq data were normalized using TPM (transcripts per million) values to ensure accurate and comparable expression levels across different tissues and developmental stages. The RNA-seq analysis was visualized using the efp tool for the transcriptomics module of the SoyMD database.

### 4.10. Plant Growth and Stress Treatments

Williams 82 soybean seeds were grown in a controlled environment greenhouse at 25 °C with a 16/8 h light/dark photoperiod. At the five-leaf stage, seedlings underwent nematode inoculations, and were subjected to drought, saline, and cold stress treatments.

Eggs of the soybean cyst nematode (SCN, *Heterodera glycines*) were incubated in a hatching buffer containing 2.99 mM ZnCl_2_ at room temperature for 72 h. The resulting stage 2 juveniles (J2) were sterilized using a solution containing 0.1 g/L HgCl2 and 0.01% sodium azide for 80 s, followed by two rinses in sterile water. The J2 SCNs were then resuspended in 0.048% sterile agarose solution for inoculation. For temperature stress experiments, seedlings were maintained at 4 °C. For drought stress, watering was withheld, and for saline stress, seedlings were treated with 0.9% NaCl.

## 5. Conclusions

In conclusion, this comprehensive study has elucidated the structural, evolutionary, and functional dynamics of the *SCPL* gene family in soybean, classifying 73 members into three distinct subgroups. Detailed analyses of gene architecture, conserved domains, and cis-acting regulatory elements highlighted the complexity and specialization of these genes in response to various environmental stresses. The findings from codon usage bias analysis and gene expression profiling under different stress conditions have further underscored the significant roles these genes play in plant stress responses. This foundational work not only advances our understanding of the *SCPL* family’s contribution to soybeans’ resilience and adaptability but also sets the stage for future research aimed at harnessing these genes for the development of stress-resistant soybean varieties. These insights provide invaluable resources for improving soybean cultivation and stress management strategies, paving the way for enhanced agricultural productivity and sustainability.

## Figures and Tables

**Table 1:** Detailed information of the putative *SCPLs* in soybean.

mRNA ID	Gene Name	Chr	Gene Location	ORF Length (bp)	Size(aa)	MW(KDa)	pI	Intron Number	Splice Variants	Subcellular Location
*Glyma.02G204700.1*	*GmSCPL1*	Chr02	38993365–39000790	1386	462	53.58417	6.76	8	1	Extracell
*Glyma.03G070300.1*	*GmSCPL2*	Chr03	16324083–16330477	1389	463	55.86286	5.67	13	1	Vacuole
*Glyma.03G090900.1*	*GmSCPL3*	Chr03	26981330–26988564	693	231	53.92149	5.34	7	1	Vacuole
*Glyma.03G125200.1*	*GmSCPL4*	Chr03	33883674–33887698	1446	482	53.43581	8.67	9	2	Vacuole
*Glyma.03G125400.1*	*GmSCPL5*	Chr03	33909174–33912239	1389	463	53.78	7.19	9	2	Vacuole
*Glyma.03G125500.1*	*GmSCPL6*	Chr03	33918983–33921895	1380	460	26.54959	4.89	9	1	Vacuole
*Glyma.03G125600.1*	*GmSCPL7*	Chr03	33929465–33932834	1386	462	33.38494	6.5	8	2	Vacuole
*Glyma.04G155900.1*	*GmSCPL8*	Chr04	36484956–36492419	1452	484	54.80313	5.73	9	1	Vacuole
*Glyma.04G200200.1*	*GmSCPL9*	Chr04	47272565–47276234	1410	470	53.0337	5.45	9	1	Vacuole
*Glyma.04G240600.1*	*GmSCPL10*	Chr04	50888492–50892907	1422	474	54.82872	5.68	9	3	Vacuole
*Glyma.06G047400.1*	*GmSCPL11*	Chr06	3584404–3590095	1416	472	42.33745	8	13	9	Vacuole
*Glyma.06G122800.1*	*GmSCPL12*	Chr06	10009186–10013239	1422	474	34.27499	7.07	9	4	Vacuole
*Glyma.06G165400.1*	*GmSCPL13*	Chr06	13723574–13727195	1413	471	56.7101	6.18	9	1	Vacuole
*Glyma.06G181700.1*	*GmSCPL14*	Chr06	15515094–15520222	1254	418	53.34864	9.14	12	5	Vacuole
*Glyma.07G192700.1*	*GmSCPL15*	Chr07	36045428–36052232	1461	487	33.0789	8.86	9	1	Vacuole
*Glyma.07G218400.1*	*GmSCPL16*	Chr07	39141266–39142853	1365	455	49.14665	9.15	0	1	Vacuole
*Glyma.07G236600.1*	*GmSCPL17*	Chr07	41820157–41824129	1446	482	55.4932	5.45	9	5	Vacuole
*Glyma.08G008700.1*	*GmSCPL18*	Chr08	682896–686265	1425	475	47.72612	5.5	9	6	Vacuole
*Glyma.08G245500.1*	*GmSCPL19*	Chr08	21195019–21201951	1416	472	51.05139	6.08	7	3	Extracell
*Glyma.08G257800.1*	*GmSCPL20*	Chr08	23162701–23166441	1386	462	59.81874	5.54	9	2	Vacuole
*Glyma.09G049500.1*	*GmSCPL21*	Chr09	4298702–4302403	1494	498	53.40589	8.32	8	2	Peroxisome/Vacuole
*Glyma.09G226700.1*	*GmSCPL22*	Chr09	45147433–45152456	1491	497	56.67716	6.07	6	1	Vacuole
*Glyma.09G249500.1*	*GmSCPL23*	Chr09	47056885–47062577	1521	507	24.22614	9.17	13	1	Peroxisome
*Glyma.10G102100.1*	*GmSCPL24*	Chr10	20236973–20240885	1395	465	51.6027	6.37	9	1	Vacuole
*Glyma.10G104300.1*	*GmSCPL25*	Chr10	22636718–22663087	1497	499	54.59697	6.28	8	2	Peroxisome/Vacuole
*Glyma.10G207300.1*	*GmSCPL26*	Chr10	43879369–43883196	1500	500	47.35157	5.82	8	1	Peroxisome/Vacuole
*Glyma.10G212500.1*	*GmSCPL27*	Chr10	44500805–44506389	1383	461	52.77113	6.72	8	2	Vacuole
*Glyma.11G099600.1*	*GmSCPL28*	Chr11	7551710–7556030	1422	474	51.0712	4.84	9	1	Vacuole
*Glyma.11G099700.1*	*GmSCPL29*	Chr11	7558753–7566979	1401	467	52.15427	8.9	9	1	Vacuole
*Glyma.11G191200.1*	*GmSCPL30*	Chr11	26434930–26442035	1377	459	54.72837	5.42	12	1	Vacuole
*Glyma.11G193800.1*	*GmSCPL31*	Chr11	26725185–26729444	1467	489	51.67685	6.02	8	5	Peroxisome/Vacuole
*Glyma.11G193900.1*	*GmSCPL32*	Chr11	26730950–26734235	1434	478	53.33445	5.98	8	1	Peroxisome/Vacuole
*Glyma.12G010100.1*	*GmSCPL33*	Chr12	718029–722960	1491	497	52.87489	6.24	6	1	Vacuole
*Glyma.12G025600.1*	*GmSCPL34*	Chr12	1844961–1853018	1449	483	51.18424	6.42	9	2	Vacuole
*Glyma.12G025900.1*	*GmSCPL35*	Chr12	1873092–1878613	1419	473	54.41969	6.89	7	1	Vacuole
*Glyma.12G083100.1*	*GmSCPL36*	Chr12	6586692–6592302	1380	460	52.64793	6.72	12	2	Vacuole
*Glyma.12G177900.1*	*GmSCPL37*	Chr12	33699331–33703039	1515	505	56.47516	5.49	8	2	Peroxisome/Vacuole
*Glyma.12G179400.1*	*GmSCPL38*	Chr12	33972888–33973824	654	218	54.03901	5.25	3	1	Vacuole
*Glyma.13G028500.1*	*GmSCPL39*	Chr13	8018267–8022397	1473	491	54.19511	6.14	7	1	Vacuole
*Glyma.13G028800.1*	*GmSCPL40*	Chr13	8182077–8185659	1596	532	51.98603	8.68	5	1	Vacuole
*Glyma.13G029200.1*	*GmSCPL41*	Chr13	8614070–8618166	1467	489	51.35516	5.49	7	1	Vacuole
*Glyma.13G075200.1*	*GmSCPL42*	Chr13	17816382–17822985	879	293	49.38054	6.42	9	3	Vacuole
*Glyma.13G183700.1*	*GmSCPL43*	Chr13	29723122–29727602	1482	494	51.78898	6.15	9	2	Vacuole
*Glyma.13G221900.1*	*GmSCPL44*	Chr13	33488305–33492501	1410	470	49.87419	6.57	13	1	Vacuole
*Glyma.13G243500.1*	*GmSCPL45*	Chr13	35288591–35293220	1437	479	55.6809	6.25	9	1	Vacuole
*Glyma.13G321200.1*	*GmSCPL46*	Chr13	41546470–41550353	1371	457	52.03607	8.72	12	1	Vacuole
*Glyma.13G321700.1*	*GmSCPL47*	Chr13	41602238–41606724	1377	459	54.5101	8.29	12	1	Vacuole
*Glyma.13G322600.1*	*GmSCPL48*	Chr13	41709896–41713941	1521	507	51.37635	6.19	8	1	Peroxisome/Vacuole
*Glyma.14G080500.1*	*GmSCPL49*	Chr14	6965564–6972041	1497	499	55.45726	5.77	7	2	Extracell
*Glyma.14G122200.1*	*GmSCPL50*	Chr14	17801426–17807621	1464	488	51.05933	5.34	9	3	Vacuole
*Glyma.15G070200.1*	*GmSCPL51*	Chr15	5375186–5380088	1449	483	55.43504	7.6	9	1	Vacuole
*Glyma.15G090300.1*	*GmSCPL52*	Chr15	6952810–6957605	1458	486	51.19083	5.13	13	2	Vacuole
*Glyma.15G156200.1*	*GmSCPL53*	Chr15	13086610–13091482	1482	494	56.10161	5.27	8	2	Peroxisome/Vacuole
*Glyma.16G082700.1*	*GmSCPL54*	Chr16	9310673–9320591	1497	499	52.89716	6.07	13	4	Peroxisome
*Glyma.16G145300.1*	*GmSCPL55*	Chr16	30604586–30608656	1482	494	54.96599	5.56	8	2	Vacuole
*Glyma.17G037000.1*	*GmSCPL56*	Chr17	2697476–2701132	1449	483	54.82264	5.96	9	1	Vacuole
*Glyma.17G073200.1*	*GmSCPL57*	Chr17	5720584–5728156	1383	461	52.40136	8.69	8	2	Vacuole
*Glyma.17G244900.1*	*GmSCPL58*	Chr17	40030818–40036322	1500	500	18.63345	4.9	7	2	Extracell
*Glyma.18G242900.1*	*GmSCPL59*	Chr18	53113341–53119592	1521	507	50.63926	6.41	13	1	Peroxisome
*Glyma.18G266700.1*	*GmSCPL60*	Chr18	55105365–55112399	1404	468	52.77681	8.51	7	1	Extracell
*Glyma.18G282200.1*	*GmSCPL61*	Chr18	56302289–56306074	1386	462	55.66993	6.65	9	1	Vacuole
*Glyma.19G128000.1*	*GmSCPL62*	Chr19	38717520–38722550	1125	375	52.91801	5.75	11	1	Vacuole
*Glyma.19G128100.1*	*GmSCPL63*	Chr19	38728037–38732422	1389	463	54.65352	4.81	10	2	Vacuole
*Glyma.19G128200.1*	*GmSCPL64*	Chr19	38739769–38743404	1383	461	52.80598	5.72	8	2	Vacuole
*Glyma.20G014900.1*	*GmSCPL65*	Chr20	1375276–1376799	903	301	43.96814	6.66	0	1	Vacuole
*Glyma.20G015100.1*	*GmSCPL66*	Chr20	1379240–1380596	1338	446	54.53818	6.36	1	1	Vacuole
*Glyma.20G015400.1*	*GmSCPL67*	Chr20	1393894–1395216	1323	441	54.39402	5.96	0	1	Vacuole
*Glyma.20G015600.1*	*GmSCPL68*	Chr20	1406934–1408603	1317	439	56.00173	5.17	0	1	Vacuole
*Glyma.20G016800.1*	*GmSCPL69*	Chr20	1545314–1546525	1182	394	54.05284	6.33	1	1	Vacuole
*Glyma.20G043400.1*	*GmSCPL70*	Chr20	7902915–7918077	1245	415	55.68712	5.76	11	3	Vacuole
*Glyma.20G056200.1*	*GmSCPL71*	Chr20	13855383–13860273	1452	484	51.60472	5.4	7	1	Vacuole
*Glyma.20G178600.1*	*GmSCPL72*	Chr20	41603247–41609549	1383	461	55.58359	5.07	8	1	Vacuole
*Glyma.U001900.1*	*GmSCPL73*	NA	NA	900	300	55.51719	5.73	0	1	Vacuole

**Table 2:** Codon usage indicators of the *SCPL* family in five different species. Abbreviations: CBI, codon bias index; Fop, frequency of optimal codons; ENC, effective number of codons; and GC3s, contents of G or C bases at the third position of the codons; and GC content, the contents of the G and C bases of the codons.

Species Name	CAI	CBI	Fop	ENC	GC3s	GC Content
*Glycine max*	0.1953835	0.089274	0.3687671	50.513836	0.3610685	0.42216438
*Arabidopsis thaliana*	0.2068823	−0.02584	0.405725	54.11863	0.40149	0.43554902
*Oryza sativa*	0.2271041	0.081895	0.4657083	48.638333	0.6486042	0.55472916
*Glycine soja*	0.1954927	0.086768	0.3701449	50.683043	0.3628841	0.42288405
*Triticum aestivum*	0.2342428	0.118742	0.4871667	48.045762	0.7024667	0.57511428

## Data Availability

Data are contained within the article.

## Supplementary Materials

The following supporting information can be downloaded at: https://www.mdpi.com/article/10.3390/ijms25126712/s1.
