# Supplementary material for: Genome-Wide Analysis of Serine Carboxypeptidase-like Genes in Soybean and Their Roles in Stress Resistance
Source: Int J Mol Sci. 2024 Jun 18;25(12):6712. doi: 10.3390/ijms25126712 (PMC11203753; doi:10.3390/ijms25126712)
Supplement: Supplementary file 1 [file ijms-25-06712-s001.zip › Figure S1-S3.pdf]

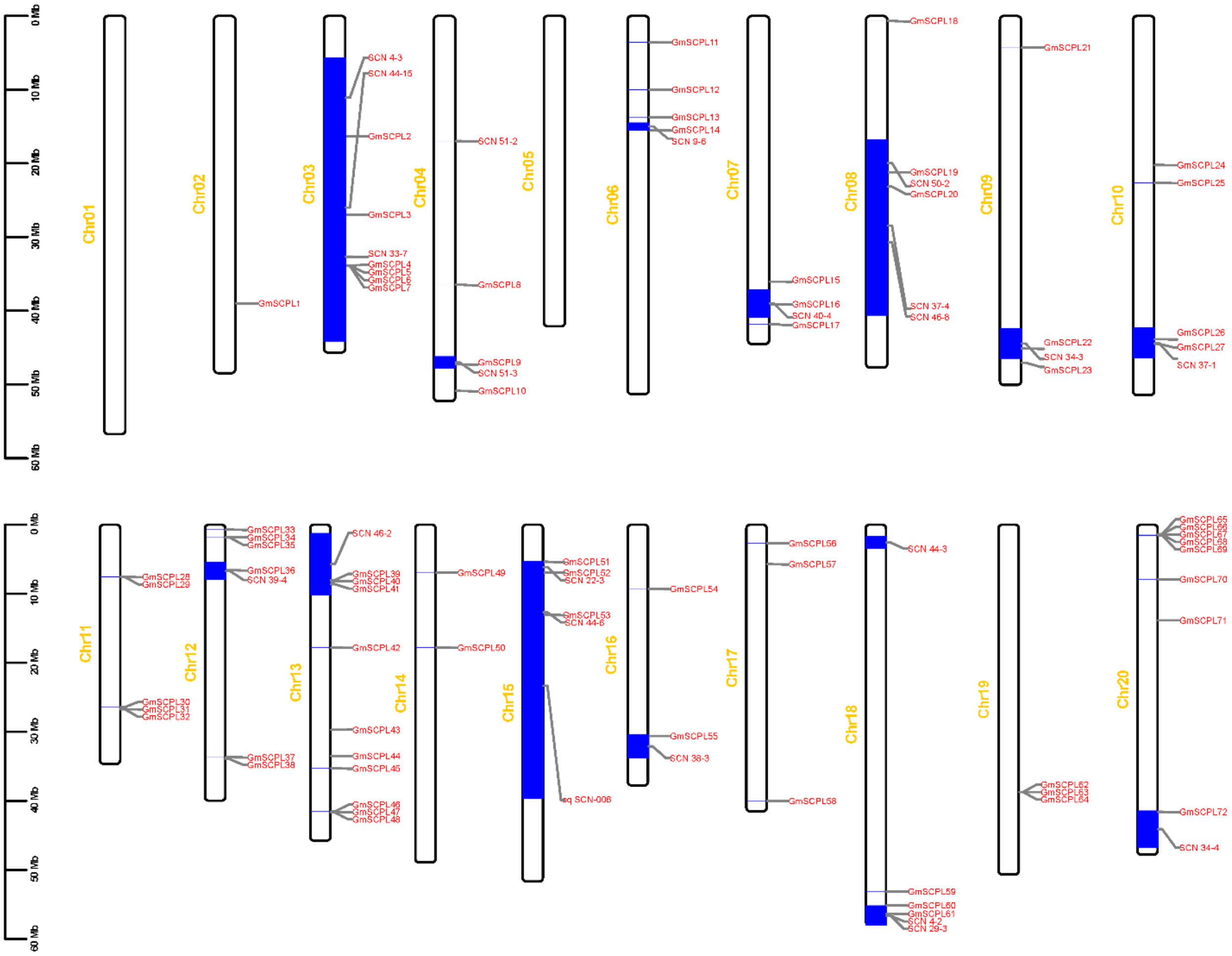

Figure S1. Chromone distribution of identified *GmSCPLs*.

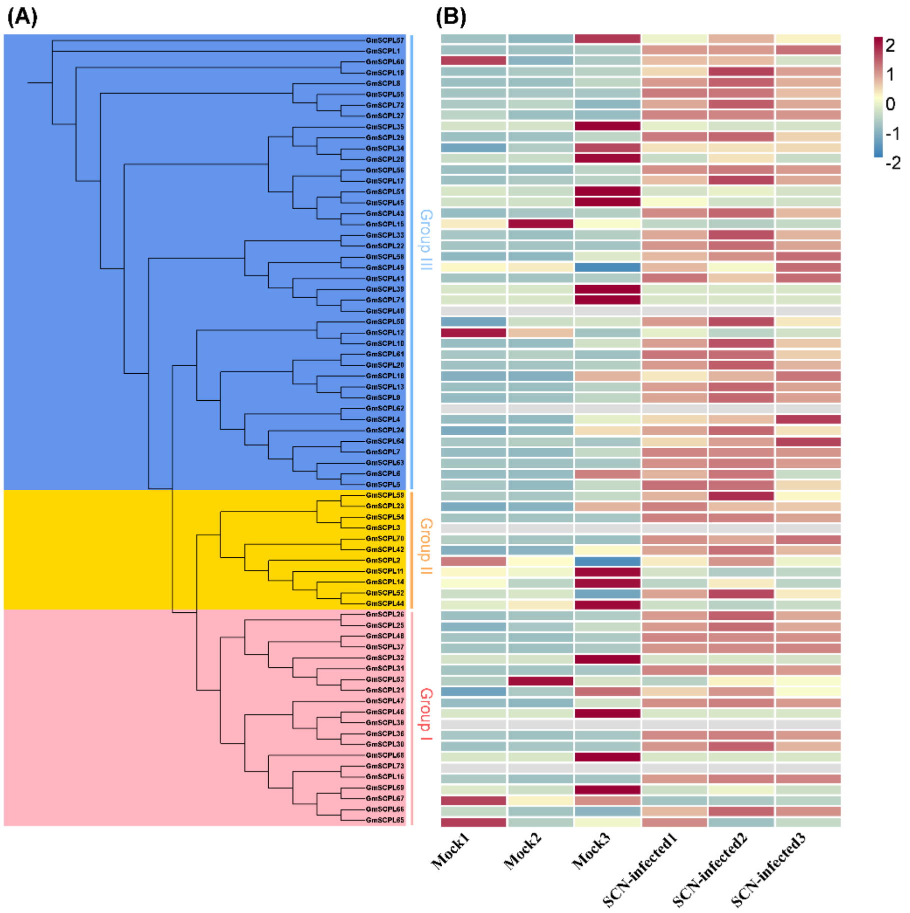

Figure S2. Heatmap analysis indicating the relative expression levels of identified *GmSCPLs* under SCN infections compared to that in mock by RNA-seq. Blue to red color representing low to high expression abundance.

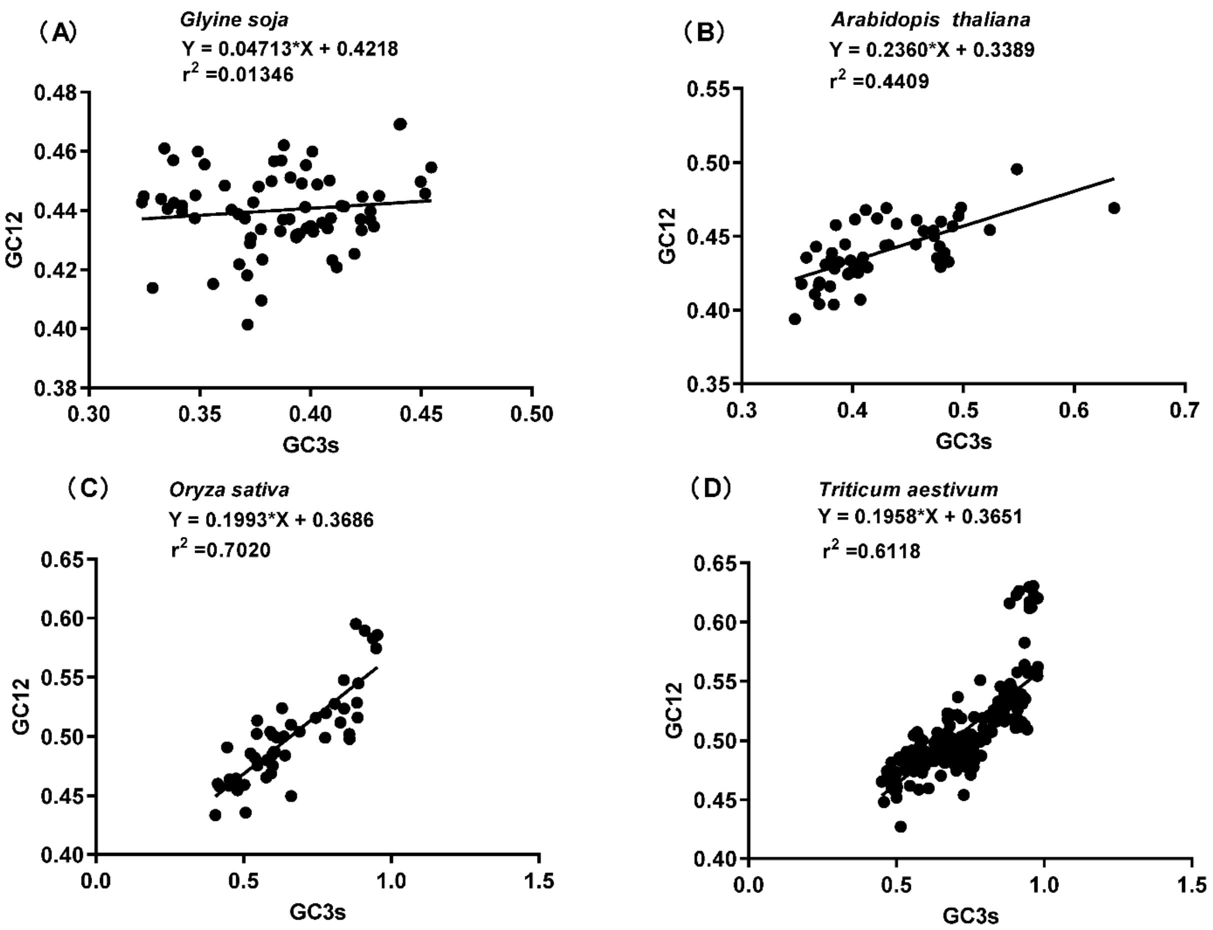

Figure S3. Neutrality plot analysis of identified *SCPL* CDS sequences in *G. soja* (A), *Arabidopsis* (B), *O.sativa* (C) *and T. aestivum* (D).
